# The SOCS2 Ubiquitin Ligase Complex Regulates Growth Hormone Receptor Levels

**DOI:** 10.1371/journal.pone.0025358

**Published:** 2011-09-29

**Authors:** Mattias Vesterlund, Fahad Zadjali, Torbjörn Persson, Michael Lund Nielsen, Benedikt M. Kessler, Gunnar Norstedt, Amilcar Flores-Morales

**Affiliations:** 1 Department of Molecular Medicine and Surgery, Karolinska Institutet, Stockholm, Sweden; 2 College of Medicine and Health Sciences, Sultan Qaboos University, Muscat, Oman; 3 Department of Neurobiology, Care Sciences and Society, Karolinska Institutet, Huddinge, Sweden; 4 Faculty of Health Sciences, Novo Nordisk Foundation Center for Protein Research, University of Copenhagen. Copenhagen, Denmark; 5 Nuffield Department of Medicine, Oxford University, Oxford, United Kingdom; Florida International University, United States of America

## Abstract

Growth Hormone is essential for the regulation of growth and the homeostatic control of intermediary metabolism. GH actions are mediated by the Growth Hormone Receptor; a member of the cytokine receptor super family that signals chiefly through the JAK2/STAT5 pathway. Target tissue responsiveness to GH is under regulatory control to avoid excessive and off-target effects upon GHR activation. The suppressor of cytokine signalling 2 (SOCS) is a key regulator of GHR sensitivity. This is clearly shown in mice where the SOCS2 gene has been inactivated, which show 30–40% increase in body length, a phenotype that is dependent on endogenous GH secretion. SOCS2 is a GH-stimulated, STAT5b-regulated gene that acts in a negative feedback loop to downregulate GHR signalling. Since the biochemical basis for these actions is poorly understood, we studied the molecular function of SOCS2. We demonstrated that SOCS2 is part of a multimeric complex with intrinsic ubiquitin ligase activity. Mutational analysis shows that the interaction with Elongin B/C controls SOCS2 protein turnover and affects its molecular activity. Increased GHR levels were observed in livers from SOCS2^−/−^ mice and in the absence of SOCS2 in *in vitro* experiments. We showed that SOCS2 regulates cellular GHR levels through direct ubiquitination and in a proteasomally dependent manner. We also confirmed the importance of the SOCS-box for the proper function of SOCS2. Finally, we identified two phosphotyrosine residues in the GHR to be responsible for the interaction with SOCS2, but only Y487 to account for the effects of SOCS2. The demonstration that SOCS2 is an ubiquitin ligase for the GHR unveils the molecular basis for its physiological actions.

## Introduction

Growth Hormone (GH) is a peptide hormone of 192 amino acids secreted from the anterior pituitary [1] that plays a key role in the regulation of intermediary metabolism and longitudinal bone growth [2], [3]. GH exerts its actions by binding to the Growth Hormone Receptor (GHR), which is present on cell membranes in several different organs and tissues. The GHR belongs to the cytokine receptor super family and signals chiefly through the Janus Kinase/Signal Transducer and Activator of Transcription (JAK/STAT) pathway. Upon GH binding the GHR is tyrosine phosphorylated by JAK2; this allows STAT5 to bind the receptor with its Src Homology 2 (SH2) domain. Next, STAT5 is phosphorylated by JAK2, dimerizes and migrates to the nucleus where it induces the expression of GH-responsive genes.

One of the genes induced by GH stimulation is the suppressor of cytokine signalling 2 (SOCS2). SOCS2 is an important negative regulator of GHR signalling as clearly demonstrated by studies of SOCS2^−/−^ mice [4]–[6]. SOCS2 deficient mice show a 40% increase in body size due to greatly enhanced postnatal growth [6]. GH-deficient, SOCS2^−/−^ mice failed to show the above-described phenotype, while treatment with exogenous GH induced excessive growth in terms of overall body weight, body and bone lengths, and the weight of internal organs and tissues. Microarray analysis on liver RNA extracts after exogenous GH administration revealed a heightened response to GH [5].

SOCS2 exerts its action through feedback inhibition of GH signalling. GH activated STAT5b binds the promoter of SOCS2 to promote expression, in turn SOCS2 binds at least two phosphorylated tyrosines on the GHR to negatively regulate JAK2 and STAT5b activation [5], [7]. The molecular basis for these actions of SOCS2 are not entirely clear and two main mechanisms of action have been proposed, one is that SOCS2 binding to the GHR blocks the association of positive regulators [5], [8]–[10]. The other hypothesis is that SOCS2 is part of an ubiquitin ligase complex targeting interacting proteins for proteasomal degradation [8], [9]. Several findings indicate that this is an important part of SOCS2 activity. SOCS2 is characterized by a SOCS-box at the C-terminal end of the protein, similarly to other ubiquitin ligases such as the Von Hippel-Lindau gene product (VHL) and SOCS1 [11]–[13]. The SOCS-box in SOCS2 interacts with Elongin B and C as revealed by the x-ray structure of the SOCS2-Elongin B/C complex [8]. In addition, another important component of SOCS ubiquitin ligases, Cullin5, has been shown to associate with SOCS2 [14]. Furthermore, deletions of the SOCS-box abrogate the inhibitory actions of SOCS2 on GH induced STAT5b activation [5]. Finally, SOCS2 was shown to be involved in the ubiquitination and proteasomal degradation of the phosphorylated proline-rich tyrosine kinase 2 (Pyk2) in Natural Killer cells in a SOCS-box dependent manner [15]. Despite all the indirect evidence, it remains undetermined whether SOCS2 actions can be explained by ubiquitination and proteasomal degradation of the key intracellular signalling components: GHR, JAK2 or STAT5b or if they involve the degradation of yet unidentified regulatory proteins. A better understanding of SOCS2 mode of action can not only shed light on the basis for its physiological functions related to GH but also to on other described activities related to inflammation, mammary gland and brain development that cannot be attributed to GH actions.

Here, we have used mutagenesis to study the influence of several domains in the SOCS2 protein on its activity. For the first time, we demonstrate a direct ubiquitin ligase activity of SOCS2 directed towards the GHR. Integrity of the SH2-domain and the SOCS-box are essential to regulate SOCS2 protein levels in mammalian cells as well as ubiquitination activity *in vitro*. Accordingly, point mutations in these domains are enough to impair the capacity of SOCS2 to downregulate GHR levels. Our studies also reveal the importance of tyrosine 487 on the GHR for its regulation by SOCS2 and thus provide a mechanistic explanation for the actions of SOCS2 on GH signalling.

## Results

### The interaction with Elongin B and C regulates SOCS2 turnover

**Figure 1:**
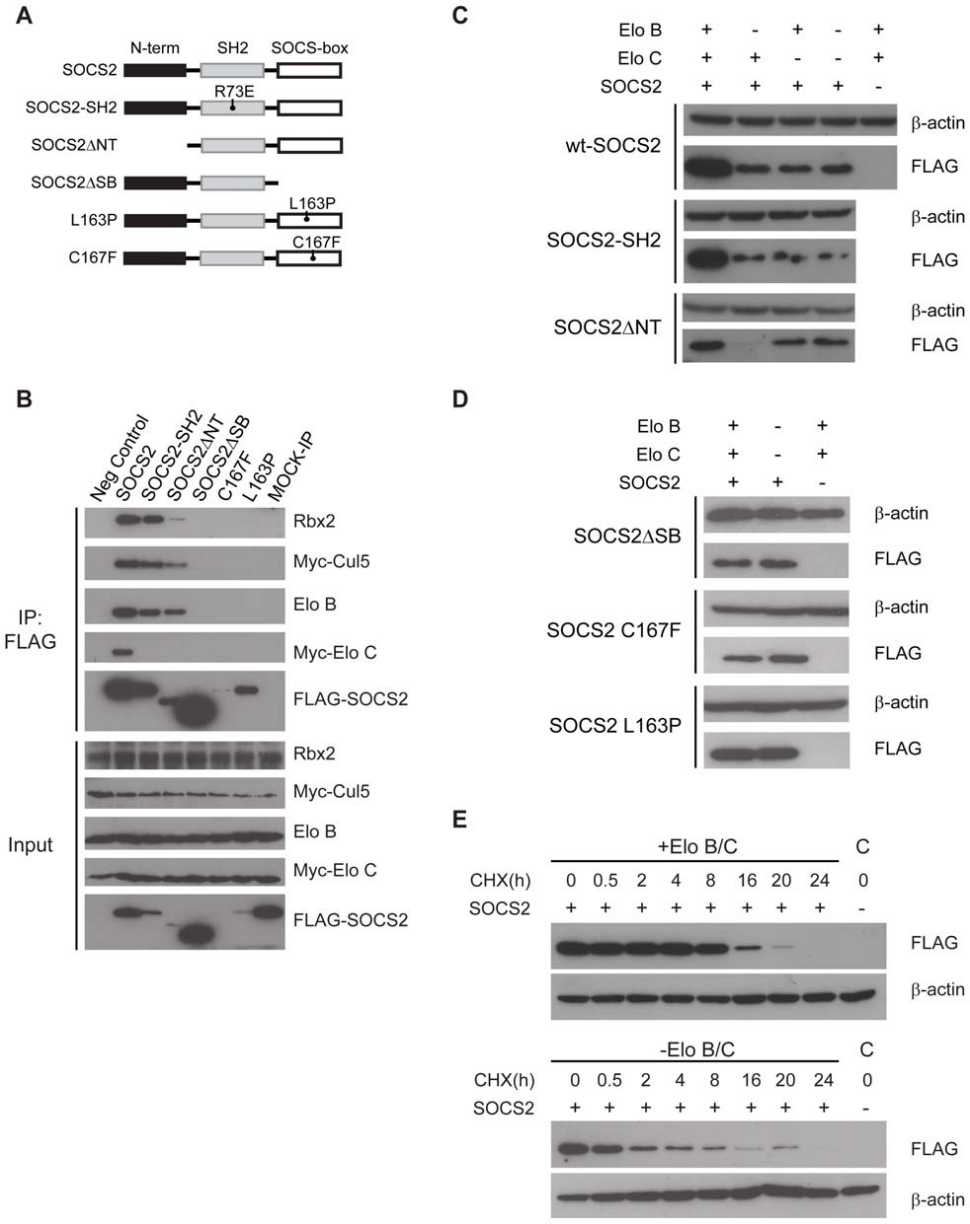
SOCS2 forms a stable complex with Elongin B and C, Cullin 5 and Rbx2 mediated by the SOCS-box. (A) Schematic of the various SOCS-constructs that were used. (B) Hek293T cells were transfected with Elongin B, Elongin C and *Myc*-Cullin5 and either an empty vector (lane 1), wildtype (lane 2 and 8) or a mutant form of FLAG-SOCS2 (lanes 3–7) as denoted in the picture. FLAG-SOCS2 and FLAG-SOCS2 mutants were immunoprecipitated with FLAG-beads (lanes 1–7), Mock-ip was immunoprecipitated with IgG. Western blots of immunoprecipitates and the lysate was performed as described in the Materials and Methods. (C) and (D) Western blot of whole cell lysate from HEK293T cells transfected with FLAG-SOCS2 and both, either or no Elongins as detailed in the figure. (E) HEK293T cells transfected with either FLAG-SOCS2 and both Elongins or FLAG-SOCS2 alone were treated with cycloheximide (100 µg/ml) for the times indicated in the figure before lysis. C denotes the control which was transfected with Empty vector instead of SOCS2.

Several studies have failed to find SOCS2 inhibitory activity towards GH activated signalling pathways despite the clear demonstration of SOCS2 activity towards GHR signalling *in vivo* [16]. One possibility to explain this discrepancy is that SOCS2 associated proteins such as Elongin B and Elongin C may need to be co-expressed with SOCS2 to achieve maximal biological activity. Therefore, we first tested whether SOCS2 forms an Elongin-Cullin-SOCS (ECS) complex in cells and how different SOCS2 domains contribute to those interactions. Expression vectors with SOCS2 cDNA containing an N-terminally located FLAG-tag were used (Figure 1A). The expression constructs were transfected into HEK293T cells and SOCS2 variants were immunoprecipitated to investigate SOCS2 interacting proteins. As shown in figure 1B, WT-SOCS2 immune precipitates were found to contain Elongin B, Elongin C, Cullin5 and Ring-box protein 2 (Rbx2) all of which are known to form part of the ECS core complex (lane 2). The interactions were confirmed by mass spectrometric analysis (data not shown). We also demonstrated that the domain responsible for the interactions is the SOCS-box. Within the SOCS-box we introduced two point mutations on well-conserved residues; L163P and C167F [8]. These residues are required for Elongin B/C binding in other SOCS-box containing proteins [17]. We did not detect Elongin B, Elongin C, Cullin5 or Rbx2 in the immunoprecipitates from the two point mutations or the SOCS-box deletion mutant (SOCSΔSB) (Figure 1B, lanes 5–7). However, the three constructs with an intact SOCS-box did co-precipitate with Elongin B, Cullin5 and Rbx2 (lanes 2–4). The SH2-domain mutant (SOCS2SH2) and the N-terminal deletion mutant (SOCS2ΔNT) did not interact with Elongin C. The reason why the SH2-domain mutant and the N-terminal deletion mutant fail to bind Elongin C is yet unclear. On the other hand, this is unlikely to be an artefact, since previous data from our lab demonstrate that a similar mutation in SOCS6 also abolishes Elongin C binding [18].

We further examined the interaction between SOCS2 and Elongin B/C. Experiments shown in Figure 1C demonstrate that SOCS2 protein content in transfected cells becomes significantly higher if Elongin C and B levels are also increased by co-transfection. The same is true for the two mutated versions of SOCS2 with an intact SOCS-box. As expected the protein levels of the mutants that lack or have a mutated SOCS-box and are not able to bind Elongin B/C are not affected by the presence of Elongin B/C (Figure 1D).

To evaluate whether or not the increased SOCS2 level in the presence of Elongins is a due to a folding or a degradation event we treated transfected cells with cycloheximide to block protein synthesis (Figure 1E). SOCS2 levels decrease faster when Elongins B/C are not co-transfected and remain stable for a longer time in the presence of Elongin B/C. From these experiments we concluded that the SOCS-box is essential for the formation of the ECS-complex and that formation stabilizes SOCS2 and reduces its degradation.

### SOCS2 displays E3 ubiquitin ligase activity *in vitro*

**Figure 2:**
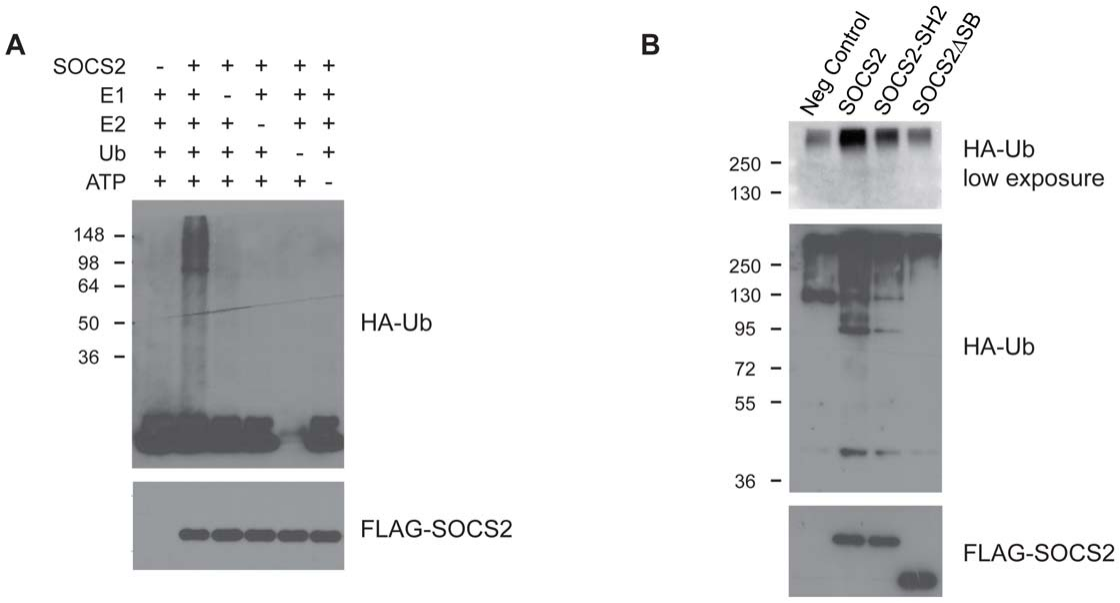
SOCS2 exhibits E3 activity in the presence of E1, E2, Ubiquitin and ATP. (A) The FLAG-tagged SOCS2-Elongin B/C complex was immunoprecipitated from HEK293T cells transfected with FLAG-SOCS2 and Elongin B/C or Empty vector and Elongin B/C. *In vitro* ubiquitination assay was performed as described in Materials and Methods except for the following: for lane 1 the assay was performed with immunoprecipitate from cells transfected with empty vector instead of SOCS2, lane 3 lacks the E1 enzyme, lane 4 lacks the E2 enzyme, lane 5 lacks Ub, lane 6 lacks ATP. Western blots of the samples were performed as described. (B) Immunoprecipitate from cells transfected with mutant variants of SOCS2 was used for the *In vitro* ubiquitination assay. Assay was performed as described in the Materials and Methods. Cells were transfected with Elongin B/C and either Empty vector (lane 1), FLAG-SOCS2 (lane 2) or a mutant form of FLAG-SOCS2 (lanes 3 and 4) as specified in the picture. Western blots of the samples were performed as described.

SOCS2s ability to bind to Elongin B and C, Cullin5 and Rbx2 led us to examine the functional relevance of this interaction. Since these four proteins are known to interact with other SOCS-box containing ubiquitin ligases [19]–[21], we wanted to investigate if the SOCS2 complex has a capacity as an ubiquitin ligase. HEK293T cells were transiently transfected with either FLAG-SOCS2 or an empty vector in concert with Elongin B and C. Cells were lysed and FLAG-tagged proteins were immunoprecipitated, and the immunoprecipitates were incubated at 30°C with human recombinant E1, UbcH5b (E2), HA-ubiquitin (Ub) and an ATP regenerating system for 30 minutes. When analyzed with a Western blot, high molecular weight signals corresponding to HA-Ub covalently attached to high molecular weight protein complexes are detected when all components of the system are present (Figure 2A, lane 2), an indication of ubiquitin ligase activity in the precipitates. We also assayed the activity of the various mutated SOCS2 constructs using the above mentioned system (Figure 2B). Our results show that the SOCS2ΔSB mutant (Figure 2B, lane 4) exhibits clearly decreased ubiquitin ligase activity as compared to the wild-type (Figure 2B, lane 2). In line with its capacity to bind Cullin5/Rbx2, we found the SOCS2-SH2 mutant also displayed ubiquitin ligase activity, although at a lower level when compared to the wild type variant. This is in line with recently published data on SOCS2 where WT-SOCS2 was shown to precipitate with ubiquitinated Pyk2 whilst SOCS2ΔSB only precipitated with the un-ubiqutinated protein [15]. We thus concluded that SOCS2 is an ubiqutin ligase and that the SOCS-box is essential for this function.

### SOCS2 regulates Growth Hormone Receptor levels

**Figure 3:**
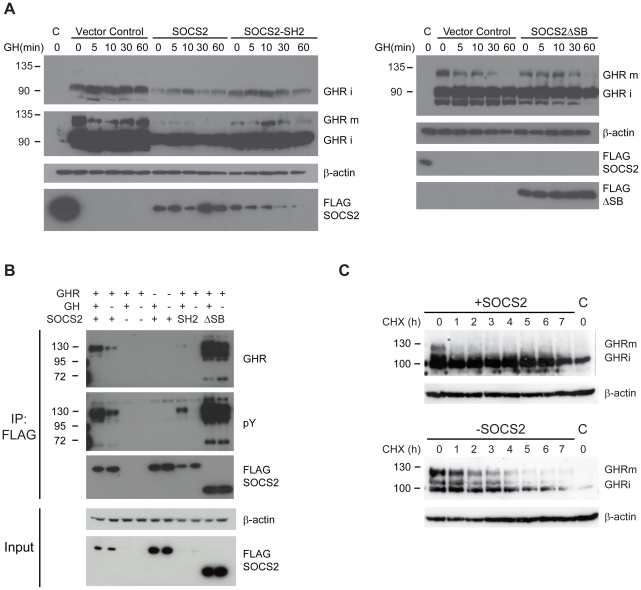
SOCS2 regulates GHR levels. (A) HEK293T cells were transfected with Elongin B/C and *Myc*-GHR and either an Empty vector, FLAG-SOCS2 or a SOCS2 mutant version. C denotes the control which was tranfected with FLAG-SOCS2 and Elongin B/C but not GHR. Cells were starved for 4 hours in Serum-free media prior to treatment. Next, cells were treated with GH for the times specified in the figure and subsequently lysed as described in the Materials and Methods. Lysates were visualized by western blot. GHR was detected with an anti-*Myc* antibody. The immature form of the GHR (i) is visible after a short exposure of the film (top panel) whilst the mature form (m) is visible after a longer exposure (2nd panel from the top). (B) Co-Immunoprecipitation of GHR. HEK293T cells were transfected with Elongin B/C, FLAG-SOCS2 or a mutant version of SOCS2 together with *Myc*-GHR (lanes 1,2 and 7–10) or only FLAG-SOCS2 and Elongins (lanes 5,6) or *Myc*-GHR alone (lanes 3,4). Cells were starved and treated with GH for 0 (−) or 10 (+) minutes prior to lysis. Lysates were immunoprecipitated as described in the materials and methods and results visualized with western blot. GHR was detected with an anti-*Myc* antibody. (C) HEK293T cells transfected with either *Myc*-GHR, FLAG-SOCS2 and Elongin B/C or GHR and Elongin B/C alone were treated with cycloheximide (100 µg/ml) and GH (2 µg/ml) for the times indicated in the figure before lysis. Cell lysates were visualized by western blot. C denotes the control which was transfected with Empty vector instead of GHR. GHR was detected with an anti-GHR antibody.

Ubiquitination is now recognized as a regulatory post-translational modification controlling protein-protein interactions and thereby influencing a variety of cellular processes. Given the inhibitory role of SOCS2 in GHR signalling and since SOCS2 exhibits ubiquitin ligase activity and can bind to phosphorylated tyrosines on the GHR [5], [10], we hypothesized that SOCS2 specifically regulates GHR turnover through proteasomal degradation. To test this hypothesis, we co-transfected SOCS2, Elongin C and Elongin B expression vectors with a GHR expression vector in HEK293T cells and analysed GHR levels by Western Blot. Prior to lysis cells were cultivated for 4 hours in DMEM without FBS and stimulated with GH for the indicated times. As shown in Figure 3A, we detected a major band and a weaker one corresponding to the immature GHR (GHR i; top left panel) and after a longer exposure a band of approximately 130 kDa corresponding to the mature, glycosylated form of the receptor (GHR m) [22], which is present in much lower concentrations. The expression of SOCS2 resulted in a significant reduction of both GHR forms, even in the absence of exogenously added GH. In contrast, cells transfected with SOCS2ΔSB did not differ from control cells regarding GHR levels. The SOCS2-SH2 mutant variant does have an effect on GHR levels but not as pronounced as the one of WT-SOCS2.

We also examined whether the mutant versions of SOCS2 were able to bind the receptor. As expected WT-SOCS2 and SOCS2ΔSB both interact with the GHR, but as figure 3B shows so does the SOCS2-SH2 construct although to a significantly lesser extent. The weak interaction is still detected which suggests that SOCS2 might be able to interact with the GHR using an extended interface, not entirely dependent on phosphotyrosine binding by the SH2-domain or alternatively involving a third partner.

The interaction between WT-SOCS2 and the GHR generally seems to be facilitated by stimulation with GH, most likely since this leads to increased tyrosine-phosphorylation of the receptor as indicated by the fact that the SOCS2-SH2 mutant binds significantly less GHR after GH stimulation. We also notice that the SOCS2-SH2 residual GHR binding activity is also GH stimulated. As for the reasons for this observation, we can only speculate but its relevance is arguably minor since the amount of GHR bound by the SOCS SH2-domain is just a minor fraction of that bound by the WT-SOCS2. Although we cannot entirely explain the unexpected behaviour of the SOCS2-SH2 mutant, we can certainly conclude from these experiments that the SH2-domain is a significant contributor to interaction of SOCS2 with the GHR in cells.

We did not observe a truly ligand dependent decrease in GHR levels in SOCS2 overexpressing cells (Figure 3A). Non stimulated cells also exhibit significantly decreased levels of the receptor. This could be related to the fact that significant amounts of GHR tyrosine phosphorylation are observed in GH untreated cells likely due to spontaneous dimerization and activation of GHR in the transfected cells.

To ascertain if the decreased GHR levels observed in SOCS2 expressing cells are due to an increase in degradation, we treated the cells with cycloheximide and examined the GHR levels in the presence and absence of SOCS2 (Figure 3C). In the absence of SOCS2 the levels of the mature GHR start to diminish after approximately 2 hours whilst mature GHR levels are undetectable after 1 hour in the presence of SOCS2 demonstrating that SOCS2 actively promotes the degradation of the mature form of the GHR.

**Figure 4:**
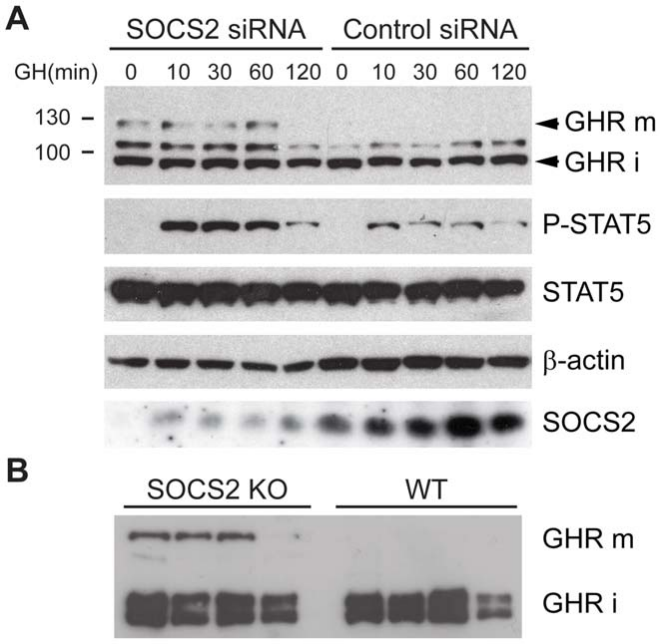
GHR levels increase in the absence of SOCS2. (A) HEK293T cells were transfected with GHR and either SOCS2 or Control siRNA. Cells were starved for 4 hours and then treated with GH for the times indicated in the picture. Cells were subsequently lysed as described in the Materials and Methods and lysates were visualized by western blot. GHR was detected with an anti-GHR antibody. (B) GHR-levels in SOCS2 KO mice. Liver lysates from SOCS2 KO mice and control mice were visualized by western blot and analyzed for GHR levels with an anti-GHR antibody.

In order to further substantiate the role of SOCS2 in the regulation of GHR protein levels we used a previously validated siRNA [23] to knockdown SOCS2 and examined the effect on GHR levels. Knockdown of SOCS2 increases the levels of GHR present in the cell, an effect that is more pronounced in the mature GHR form. Consequently, the phosphorylation of STAT5 upon GH treatment is increased in SOCS2 siRNA treated cells (Figure 4A). Similar results were observed when liver from SOCS2^−/−^ mice were analyzed. The levels of GHR were also enhanced in SOCS2^−/−^ mice livers as compared to WT controls (Figure 4B). In conclusion, in the absence of SOCS2 the mature GHR levels and downstream GHR signalling are increased (Figure 4A).

From these results we ascertained that SOCS2 indeed regulates the level of GHR present in the cell and that this is an effect dependent on a functional SOCS-box (Figure 3A). The primary effect of SOCS2 seems to be increased degradation of the mature signalling form of GHR (Figure 3C).

### SOCS2 targets the GHR for proteasomal degradation

**Figure 5:**
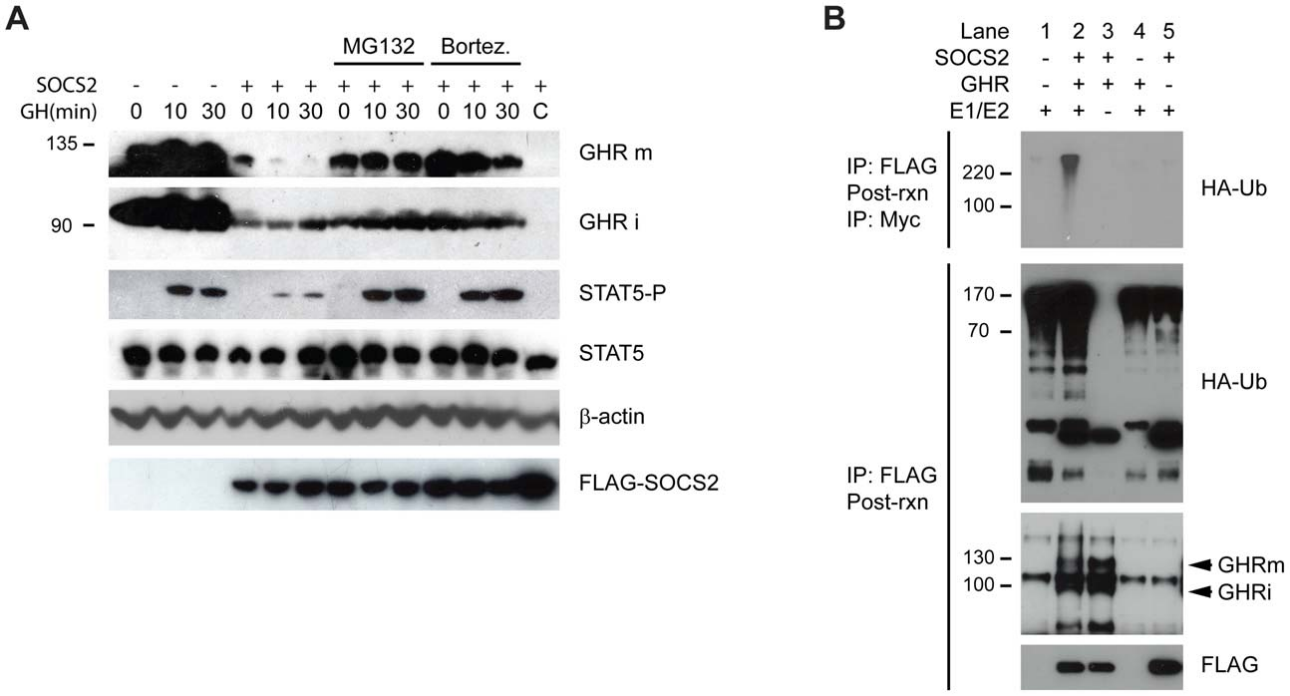
SOCS2 targets the GHR for proteasomal degradation. (A) Cells were transfected with GHR and Elongin B/C alone or in concert with FLAG-SOCS2. C denotes the control which was transfected with FLAG-SOCS2 and Elongin B/C. Cells were starved for 2 hours then treated with the indicated proteasomal inhibitors for 2 hours (20 µM MG132 or 5 nM Bortezomib (*Bortez.*)) followed by GH treatment as indicated. Cells were subsequently lysed as described in the Materials and Methods and lysates were visualized by western blot. GHR was detected with an anti-*Myc* antibody. (B) SOCS2 ubiquitinates the GHR i*n vitro*. Cells were transfected with Elongin B/C and either Empty vector (Lane 1), FLAG-SOCS2 (Lane 5), GHR (Lane 4) or both (Lane 2 and 3). Cells were starved, treated with 5 nM Bortezomib for 2 hours and GH for 10 min followed by lysis. After lysis FLAG-tagged SOCS2 and proteins binding to it were immunoprecipitated with FLAG beads. In vitro Ub reaction was performed with E1/E2 (All except lane 3), ATP-system, Ub-aldehyde, HA-Ub and normal Ub in 100 µl. After the reaction proteins were eluted from the beads with a FLAG-peptide (100 µg/ml) and immunoprecipitated a scond time with α-Myc Ab. After IP, samples were boiled and visualized by western blot. GHR was detected with an anti-GHR antibody. Top three panels: samples post reaction. Bottom panel: samples after second IP.

It has been reported earlier that the proteasome and the ubiquitin system is involved in the endocytosis of GHR [24]. Furthermore SOCS2 has been shown to regulate the levels of Pyk2 and to increase its ubiquitination [15]. To elucidate if SOCS2 regulates GHR availability by a proteasomal pathway, we treated cells transfected with SOCS2, Elongins and GHR with proteasome inhibitors MG132 and Bortezomib (Figure 5A). Both proteasome inhibitors counteracted the reducing effect of SOCS2 on receptor levels. Again, the effect is mainly noticeable for the mature receptor. This suggests that SOCS2s regulation of GHR levels is dependent on a functional proteasome.

To further examine the mechanism by which SOCS2 affects the GHR we performed an *in vitro* ubiquitination assay with SOCS2 that had been co-precipitated with GHR. After the completion of the ubiquitination assay, SOCS2 was eluted from the beads and the growth hormone receptor was immunoprecipitated with a myc-antibody and its ubiquitination level was examined by probing with an anti HA-tag antibody. As shown in Figure 5B, high molecular weight Myc-GHR/HA-Ubiquitin conjugates are more abundant when SOCS2 and GHR are co-precipitated and all components of the *in vitro* ubiquitination reaction are present (lane 2). This suggests that SOCS2 is capable of ubiquitinating GHR *in vitro* and thus targeting it for proteasomal degradation.

### SOCS2 effects are mediated through Tyr487 on the GHR

**Figure 6:**
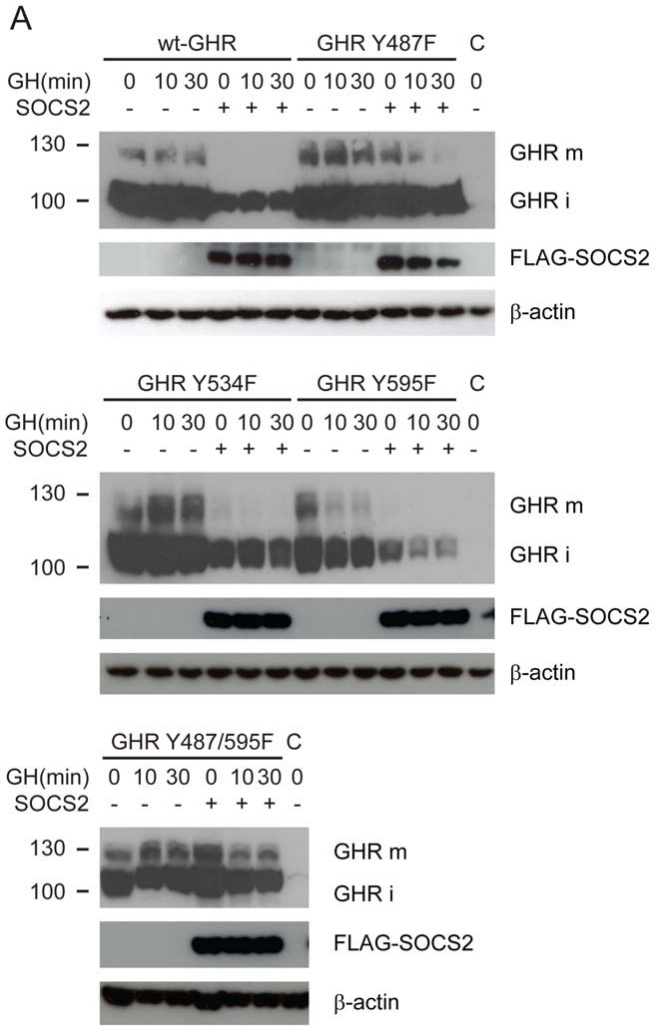
SOCS2 downregulation of GHR-levels is mediated by Y487. (A) HEK293T cells were transfected with Elongin B/C, Myc-GHR or a mutant version of the receptor alone or in concert with FLAG-SOCS2 as specified in the figure. C denotes the control which was transfected with Empty Vector. Cells were starved for 4 hours, treated with GH for the times indicated in the figure and lysed. Lysates were visualized by western blot as detailed in the Materials and Methods. GHR was detected with an anti-*Myc* antibody.

Since SOCS2 contains an SH2-domain capable of binding phosphorylated tyrosines we used mutational analysis to determine which tyrosines on the GHR are important for SOCS2s regulation of the receptor. It has previously been reported that the SH2-domain of SOCS2 binds to phosphopeptides corresponding to the residues Y487 and Y595 on the GHR [5], [10]. We employed four different GHR constructs (Y487F, Y534F, Y595F and Y487/595F) and assayed the effect of SOCS2 on the levels of these mutant receptors (Figure 6A). The levels of two of the mutants are clearly less affected by the presence of SOCS2; namely Y487F and the double mutant Y487/595F. This demonstrates that tyrosine 487 is of great functional importance for SOCS2 mediated regulation of GHR. This is in line with previous work from our lab that has demonstrated that STAT5b signalling for the Y487F and Y487/595F GHR mutants are unaffected by increasing concentrations of SOCS2 [5]. Surprisingly, the SOCS2 SH2-domain has a higher affinity for peptides corresponding to phosphorylated Tyr595 than for those containing phosphorylated Tyr487 [5] but these differences in affinity doesn't seem relevant for the function of SOCS2 in the control of GHR levels.

## Discussion

In this work we provide a mechanistic explanation for how SOCS2 acts as a negative regulator of GHR signalling [5], [10], [25]. SOCS2 assembles a canonical ECS complex with intrinsic ubiquitin ligase activity through interactions with Cullin5, Rbx2, and Elongins C and B. The SOCS2 ubiquitin ligase complex has the ability to ubiquitinate the GHR *in vitro* and downregulate GHR in a proteasomally dependent manner. The downregulatory activity of SOCS2 on the GHR is mediated by both a functional SOCS-box and by its interaction with Tyr487 in the intracellular domain of the GHR.

The structure of Elongin B/C in complex with the SOCS-box domain of SOCS2, SOCS3 and VHL has been deciphered and predict a very strong affinity between Elongin C and the SOCS-box which has lead to the proposal that Elongin B/C interaction is important for the correct folding and expression of the SOCS proteins [8], [26]–[28]. Our data showing the reduced expression of the SOCS2 L163P and C167F SOCS-box and the R73E SH2-domain mutants, which are defective in Elongin C interaction lend support to these hypotheses and is in line with previous data from studies with VHL tumour suppressor SOCS-box point mutations [29]. However, the entire deletion of the C-terminal end of SOCS2 (SOCS2ΔSB) resulted in dramatically increased levels of SOCS2 as compared to the WT. For the SOCS-box deficient SOCS2ΔSB, the stability is not influenced in any way by the presence of the two Elongins. We can also note that its protein level remains constantly high for all experiments. The simplest explanation for this observation is that the SOCS2 C-terminus also contains signals for degradation which reside outside of the SOCS-box. A possibility is that Cullin5, whose binding motif in SOCS2 lays outside the Elongin B/C binding domain [30] mediates the autoubiquitination and degradation of SOCS2. Indeed, autoubiquitination of adaptor subunits has been described for the Vif-Elongin B/C-Cullin5 complex [31] and the structurally related Skp-Cullin-F-box family of E3 ligases [32]. However, we have not detected the appearance of SOCS2-ubiquitin complexes during in vitro ubiquitination assays and further studies are needed before a definite conclusion can be drawn.

We have demonstrated for the first time that the SOCS2 complex exhibits ubiquitin ligase activity *in vitro*, specifically towards the GHR. The analysis of mutant variants of SOCS2, confirm that SOCS2 activity is dependent on the interaction with Elongin B/C and Cullin5/Rbx2 through its C-terminus. The SH2-mutant showed ubiqutin ligase activity, which is in line with the finding that this mutant retains the ability to interact with Cullin5 and Rbx2 although it shows reduced binding to Elongin C. This may explain the residual ubiqutin ligase activity observed in the *in vitro* ubiquitination assays for this mutant since the ring finger protein Rbx2 is considered an essential component of the enzymatic reaction leading to the transfer of ubiquitin moieties to the target proteins. We have also shown that overexpression of SOCS2 downregulates GHR levels, especially the mature form of the receptor. This downregulation is not evident when the SOCS2ΔSB mutant is overexpressed, even though this mutant retains the ability to bind to the receptor. This demonstrates that the ability to form a complete E3 ligase complex is essential for SOCS2 ability to regulate GHR levels. However, the SH2-domain mutant retains both a slight ability to bind to the receptor and has an effect on GHR levels; this suggests that the SH2-domain might not be the only domain involved in GHR binding. The existence of target binding motifs outside the SH2-domain has also been demonstrated for SOCS6, where an additional interacting domain found in the N-terminus is involved in the recognition and ubiquitination of the p56*^lck^* tyrosine kinase [33].

Previous publications have shown that the proteasome plays an important role in GHR endocytosis and degradation [34]. We also investigated the effect of proteasome inhibitors on GHR levels and found that these counteract SOCS2 and lead to increased GHR levels. Together with the evidence that SOCS2 is able to ubiquitinate the GHR *in vitro*, it would suggest that SOCS2 mediated ubiquitination leads to endocytosis and proteasomal degradation of the receptor. On the other hand SOCS2 is not the only ubiquitin ligase acting on the GHR. Van Kerkhof et al. have showed how the ubiquitin ligase ß-TrCP is involved in the regulation of GHR endocytosis and degradation in a ligand independent manner [35]. We have also noted in our experiments that siRNA based ß-TrCP downregulation increase GHR levels. In this background, SOCS2 retains its ability to downregulate GHR levels, arguing for an independent effect of SOCS2 on GHR turnover (data not shown). The dependency of SOCS2 mediated downregulation on GHR Tyr487 phosphorylation, add support for the existence of parallel pathways of GHR ubiquitination driven by ß-TrCP and SOCS2. The fact that SOCS2 seems to preferentially regulate mature, activated receptors seem to be a crucial aspect of its physiological actions as manifested in the phenotypic gigantism observed in SOCS2^−/−^ mice in comparison with the normal growth rate reported for ß-TrCP1^−/−^ mice [4], [36].

Several studies indicate that in addition to its actions on GHR signalling, SOCS2 also has important actions in neuron development [37], the regulation of metabolism [38], mammary gland development and cancer [39] as well as the immune response [39]–[41]. Although it seems unlikely that the various functions of SOCS2 are all GHR mediated, the wide tissue distribution of SOCS2 [42] together with our demonstration that SOCS2 controls GHR levels invite a further exploration of these phenotypes to assess the possible role of GHR signalling. Likewise, future studies of SOCS2 mechanisms of action should include an assessment of its ubiquitin ligase activity as an end-point measurement. Already, several polymorphisms of the SOCS2 gene has been identified in humans, one linked to height [43] while the other one is linked to type 2 diabetes [44]. Our results suggest that disorders associated to SOCS2 lack of function may possibly be treated by GHR antagonists, one of which is already in clinical use. This is an exiting opportunity that awaits further validation of the role of SOCS2 in human disease.

## Materials and Methods

### Ethical Statement

All animal experimentations described in this work were conducted in accordance with European and Spanish laws and regulations (European convention 123, on the use and protection of vertebrate mammals used in experimentation and other scientific purposes. Spanish R.D 223/88 and O.M. 13-10-89 of the Ministry of Agricultural, Food, and Fisheries on the protection and use of animals in scientific research and internal biosafety and bioethics guidelines). The study was approved by the Ethical Committee of the University of Las Palmas de Gran Canaria, to Professor Leandro Fernández-Pérez (project ID: MIICINN-SAF2006-07824SAF2006).

### Animal experiments

SOCS2^−/−^ mice (C57BL/6J) have been described [6]. Mice were housed under controlled temperature (23°C) and lighting (12 hr-light and 12 hr-dark cycles) with free access to water and food. Male SOCS2^−/−^ and wild-type (WT) mice between 27–28 weeks old were allocated into groups (3–7 mice per cage) and were fed a standard control diet. Mice were sacrificed by CO_2_ gas followed by cervical dislocation. Livers were excised and snap-frozen in liquid nitrogen before being stored at −80°C. 20–50 mg of liver tissue was homogenized in 1mL ice-cold buffer containing: 50 mM Tris HCl pH 7.5/150 mM NaCl/5 mM EDTA/0.5% Igepal-40/1 mM Na_3_VO_4_/20 mM NaF/1 mM dithiothretiol (DTT)/1 mM phenylmethanesulphonylfluoride (PMSF)/1× Cocktail inhibitor (Complete mini, Roche)/1× phosphatase inhibitor cocktail 2 (Sigma). Homogenates were cleared by centrifugation at 14,000× *g* for 15 minutes at 4°C.

### Cell culture

Human Embryonic Kidney cells (HEK 293T) were obtained from the American Type Culture Collection (ATCC) and were cultured in Dulbecco's Modified Eagle's Medium (DMEM) supplemented with 10% Foetal Bovine Serum (FBS) (Gibco), 100 U/ml penicillin and 100 µg/ml streptomycin (PAA). Cells were grown in the presence of 5% CO_2_ at 37°C. Cells were lysed in 50 mM Tris HCl, pH 7.5/150 mM NaCl/5 mM EDTA/0.5% Igepal-40/1 mM Na_3_VO_4_/20 mM NaF/1 mM DTT/1 mM PMSF/1× Cocktail inhibitor (Complete mini, Roche). Cell debris was removed by centrifugation at 14,000× *g* for 15 minutes at 4°C. GH treatment concentration was 2 µg/ml unless otherwise specified.

### Transfection

Amounts of between 3–10 µg of plasmid DNA was used for transfections. Transfections were carried out by employing SuperFect (Qiagen) according to the manufacturer's directions. SOCS2, Elongin C and Elongin B were transfected at a 2∶1∶1 ratio. Samples lacking any of the components were brought to the same amount of DNA by addition of an empty vector.

### Plasmids and siRNA

Plasmids used for transfections were described earlier [5]. The SOCS2 and GHR expression vectors were kind gifts by Prof. Douglas Hilton and Prof. Nils Billestrup, respectively. All versions of SOCS2 and Elongin B and C were in the pEF-BOS vector [45]. Constructs used were: FLAG-SOCS2 (SOCS2), pEF-BOS (Empty vector), SOCS2-FLAG with a point mutation in the SH2-domain at R73E (SOCS2SH2), FLAG- SOCS2 lacking the 37 amino acid N-terminus (SOCS2ΔNT), FLAG-SOCS2 lacking the 39 amino acid C-terminus corresponding to the SOCS-box (SOCSΔSB), FLAG-SOCS2 with a point mutation in the SOCS-box at L163P (L163P), FLAG-SOCS2 with a point mutation in the SOCS-box at C167F (C167F), *Myc*-Growth Hormone Receptor (GHR), GHR-vectors with point mutations at Y487F (487), Y534F (534), Y595F (595) and Y487/595F (487/595), Elongin B (Elongin B) and *Myc*-Elongin C (Elongin C). 3 µg (60 mm plate) or 9 µg (100 mm plate) of siRNA was used for knock-down experiments. siRNA directed against SOCS2 and Control siRNA were purchased from QIAGEN and described previously [23].

### Mutagenesis

In order to introduce point mutations in the SOCS2-FLAG construct described above the Stratagene QuikChange II Site-directed mutagenesis kit was used according to the manufacturer's instructions. Two mutants were created; the L163P and the C167F, both described above. The primers used were 5′-CATCAGCACCCACTCCCCAGCATTTCTGTCGAC-3′, 5′-GTCGACAGAAATGC-TGGGGAGTGGGTGCTGATG-3′, 5′-CCCACTCTGCAGCATTTCTTTCGACTCGCC-3′ and 5′-GGCGAGTCGAAAGAAATGCTGCAGAGTGGG-3′.

### Western blot

Whole cell lysates or immunoprecipitates from HEK293T cells were separated in SDS/PAGE gels and transferred to polyvinylidenediflouride (PVDF) membranes (Millipore). After blotting membranes were blocked in 5% non-fat skim milk or BSA (Sigma) in Tris-Buffered Saline (TBS) containing 0,1% Tween 20. Membranes were incubated with one or more of the following antibodies as specified in the figures and figure legends; Horse-radish peroxidase (HRP)-conjugated anti-Flag M2 (Sigma), HRP-conjugated anti-Hemagglutinin Tag (HA) (Sigma), mouse anti-β-actin (Santa Cruz),rabbit anti-phosphotyrosine (pY) (Cell Signaling), rabbit anti-Rbx2 (Santa Cruz), goat anti-Elongin B (Santa Cruz), goat anti-pY699-STAT5 (Cell Signaling), goat anti-STAT5 (Cell Signaling), mouse anti-Myc (Santa Cruz), mouse anti-GHR (Santa Cruz), rabbit anti-SOCS2 (Cell Signaling) followed, when necessary with incubation with the appropriate HRP-conjugated secondary antibody (Santa Cruz). Membranes were visualized with the ECL Western blotting detection system (Pierce) according to the manufacturer's instruction. Both SDS/PAGE and blotting was carried out in Surelock XCell (Invitrogen).

### Immunoprecipitation

HEK 293T cells were cultivated in Ø100 mm plates and transfected with 8–10 µg of DNA as described above. Cells were lysed in 50 mM Tris HCl, pH 7.5/150 mM NaCl/5 mM EDTA/0.5% Igepal-40/1 mM Na_3_VO_4_/20 mM NaF/1 mM PMSF/1× Cocktail inhibitor (Roche). Lysates were centrifuged at 14,000× *g* for 15 minutes at 4°C to remove cell debris. Supernatants from identical samples were pooled and rotated with 10 µl sepharose beads (Amersham) per sample for 1 h at 4°C to remove proteins binding to sepharose. Samples were spun down for 2 minutes at 1000 rpm at 4°C. For immunoprecipitation with anti FLAG-antibody supernatants were rotated with 10 µl Flag-conjugated agarose beads (Sigma) per sample for 1 h at 4°C. For immunoprecipitation with anti-Myc antibody, supernatants were rotated with 2 µg of antibody over night at 4°C. After supernatants had been removed the beads were washed in Equalizing buffer; 50 mM Tris HCl, pH 7.5/150 mM NaCl/5 mM EDTA.

### *In vitro* ubiquitination assay

Following immunoprecipitation beads were washed three times in HEB buffer; 20 mM Tris HCl, pH 7.5/5 mM KCl/1.5 mM MgCl_2_/1 mM DTT/10 µM MG132. Beads were resuspended in 20 µl HEB buffer per sample. The packed and washed Flag-conjugated beads containing FLAG-WT or FLAG-mutant SOCS2 complexes were incubated with 1 µl E1 (0.5 µg/µl His_6_-tagged, human recombinant, Biomol)/1.5 µl E2 (1 µg/µl His_6_-tagged, human recombinant, Biomol)/8 µl 200 mM Ub/5 µl 100 mM HA-Ub/8 µl ATP regenerating system (10 mM ATP/20 mM Tris HCl, pH 7.5/10 mM MgCl_2_/300 mM creatinine phosphate (Roche)/0.5 ng/µl rabbit creatinine kinase (Calbiochem)) at 30°C for 30 minutes. The reaction was carried out in HEB buffer and the total reaction volume was 100 µl. Reactions were either stopped by the addition of 4×SDS sample buffer (Invitrogen) followed by heating to 70°C or by the addition of 20 mM EDTA. For samples stopped with EDTA, proteins bound to the beads were eluted by a 2 hour incubation with FLAG-peptide (100 µg/ml) followed by immunoprecipitation with anti-*Myc* antibody as described above.

## References

[1] Deng L, He K, Wang X, Yang N, Thangavel C (2007). Determinants of growth hormone receptor down-regulation.. Mol Endocrinol.

[2] Tritos NA, Biller BM (2009). Growth hormone and bone.. Curr Opin Endocrinol Diabetes Obes.

[3] Ahmed SF, Farquharson C (2010). The effect of GH and IGF1 on linear growth and skeletal development and their modulation by SOCS proteins.. J Endocrinol.

[4] Greenhalgh CJ, Bertolino P, Asa SL, Metcalf D, Corbin JE (2002). Growth enhancement in suppressor of cytokine signaling 2 (SOCS-2)-deficient mice is dependent on signal transducer and activator of transcription 5b (STAT5b).. Mol Endocrinol.

[5] Greenhalgh CJ, Rico-Bautista E, Lorentzon M, Thaus AL, Morgan PO (2005). SOCS2 negatively regulates growth hormone action in vitro and in vivo.. J Clin Invest.

[6] Metcalf D, Greenhalgh CJ, Viney E, Willson TA, Starr R (2000). Gigantism in mice lacking suppressor of cytokine signalling-2.. Nature.

[7] Vidal OM, Merino R, Rico-Bautista E, Fernandez-Perez L, Chia DJ (2007). In vivo transcript profiling and phylogenetic analysis identifies suppressor of cytokine signaling 2 as a direct signal transducer and activator of transcription 5b target in liver.. Mol Endocrinol.

[8] Bullock AN, Debreczeni JE, Edwards AM, Sundstrom M, Knapp S (2006). Crystal structure of the SOCS2-elongin C-elongin B complex defines a prototypical SOCS box ubiquitin ligase.. Proc Natl Acad Sci U S A.

[9] Johnston JA (2004). Are SOCS suppressors, regulators, and degraders?. J Leukoc Biol.

[10] Uyttendaele I, Lemmens I, Verhee A, De Smet AS, Vandekerckhove J (2007). Mammalian protein-protein interaction trap (MAPPIT) analysis of STAT5, CIS, and SOCS2 interactions with the growth hormone receptor.. Mol Endocrinol.

[11] Zhang JG, Metcalf D, Rakar S, Asimakis M, Greenhalgh CJ (2001). The SOCS box of suppressor of cytokine signaling-1 is important for inhibition of cytokine action in vivo.. Proc Natl Acad Sci U S A.

[12] Maxwell PH, Wiesener MS, Chang GW, Clifford SC, Vaux EC (1999). The tumour suppressor protein VHL targets hypoxia-inducible factors for oxygen-dependent proteolysis.. Nature.

[13] Cockman ME, Masson N, Mole DR, Jaakkola P, Chang GW (2000). Hypoxia inducible factor-alpha binding and ubiquitylation by the von Hippel-Lindau tumor suppressor protein.. J Biol Chem.

[14] Lavens D, Montoye T, Piessevaux J, Zabeau L, Vandekerckhove J (2006). A complex interaction pattern of CIS and SOCS2 with the leptin receptor.. J Cell Sci.

[15] Lee SH, Yun S, Piao ZH, Jeong M, Kim DO (2010). Suppressor of cytokine signaling 2 regulates IL-15-primed human NK cell function via control of phosphorylated Pyk2.. J Immunol.

[16] Ridderstrale M, Amstrup J, Hilton DJ, Billestrup N, Tornqvist H (2003). SOCS-3 is involved in the downregulation of the acute insulin-like effects of growth hormone in rat adipocytes by inhibition of Jak2/IRS-1 signaling.. Horm Metab Res.

[17] Kamura T, Burian D, Yan Q, Schmidt SL, Lane WS (2001). Muf1, a novel Elongin BC-interacting leucine-rich repeat protein that can assemble with Cul5 and Rbx1 to reconstitute a ubiquitin ligase.. J Biol Chem.

[18] Zadjali F, Pike AC, Vesterlund M, Sun J, Wu C (2011). Structural basis for c-KIT inhibition by the suppressor of cytokine signaling 6 (SOCS6) ubiquitin ligase.. J Biol Chem.

[19] Heuze ML, Guibal FC, Banks CA, Conaway JW, Conaway RC (2005). ASB2 is an Elongin BC-interacting protein that can assemble with Cullin 5 and Rbx1 to reconstitute an E3 ubiquitin ligase complex.. J Biol Chem.

[20] Kohroki J, Nishiyama T, Nakamura T, Masuho Y (2005). ASB proteins interact with Cullin5 and Rbx2 to form E3 ubiquitin ligase complexes.. FEBS Lett.

[21] Kamizono S, Hanada T, Yasukawa H, Minoguchi S, Kato R (2001). The SOCS box of SOCS-1 accelerates ubiquitin-dependent proteolysis of TEL-JAK2.. J Biol Chem.

[22] van den Eijnden MJ, Strous GJ (2007). Autocrine growth hormone: effects on growth hormone receptor trafficking and signaling.. Mol Endocrinol.

[23] Hu J, Winqvist O, Flores-Morales A, Wikstrom AC, Norstedt G (2009). SOCS2 influences LPS induced human monocyte-derived dendritic cell maturation.. PLoS One.

[24] Strous GJ, dos Santos CA, Gent J, Govers R, Sachse M (2004). Ubiquitin system-dependent regulation of growth hormone receptor signal transduction.. Curr Top Microbiol Immunol.

[25] Hansen JA, Lindberg K, Hilton DJ, Nielsen JH, Billestrup N (1999). Mechanism of inhibition of growth hormone receptor signaling by suppressor of cytokine signaling proteins.. Mol Endocrinol.

[26] Stebbins CE, Kaelin WG, Pavletich NP (1999). Structure of the VHL-ElonginC-ElonginB complex: implications for VHL tumor suppressor function.. Science.

[27] Botuyan MV, Koth CM, Mer G, Chakrabartty A, Conaway JW (1999). Binding of elongin A or a von Hippel-Lindau peptide stabilizes the structure of yeast elongin C.. Proc Natl Acad Sci U S A.

[28] Babon JJ, Sabo JK, Soetopo A, Yao S, Bailey MF (2008). The SOCS box domain of SOCS3: structure and interaction with the elonginBC-cullin5 ubiquitin ligase.. J Mol Biol.

[29] Schoenfeld AR, Davidowitz EJ, Burk RD (2000). Elongin BC complex prevents degradation of von Hippel-Lindau tumor suppressor gene products.. Proc Natl Acad Sci U S A.

[30] Babon JJ, Sabo JK, Zhang JG, Nicola NA, Norton RS (2009). The SOCS box encodes a hierarchy of affinities for Cullin5: implications for ubiquitin ligase formation and cytokine signalling suppression.. J Mol Biol.

[31] Mehle A, Goncalves J, Santa-Marta M, McPike M, Gabuzda D (2004). Phosphorylation of a novel SOCS-box regulates assembly of the HIV-1 Vif-Cul5 complex that promotes APOBEC3G degradation.. Genes Dev.

[32] Kus BM, Caldon CE, Andorn-Broza R, Edwards AM (2004). Functional interaction of 13 yeast SCF complexes with a set of yeast E2 enzymes in vitro.. Proteins.

[33] Choi YB, Son M, Park M, Shin J, Yun Y (2010). SOCS-6 negatively regulates T cell activation through targeting p56lck to proteasomal degradation.. J Biol Chem.

[34] van Kerkhof P, Govers R, Alves dos Santos CM, Strous GJ (2000). Endocytosis and degradation of the growth hormone receptor are proteasome-dependent.. J Biol Chem.

[35] van Kerkhof P, Putters J, Strous GJ (2007). The ubiquitin ligase SCF(betaTrCP) regulates the degradation of the growth hormone receptor.. J Biol Chem.

[36] Nakayama K, Hatakeyama S, Maruyama S, Kikuchi A, Onoe K (2003). Impaired degradation of inhibitory subunit of NF-kappa B (I kappa B) and beta-catenin as a result of targeted disruption of the beta-TrCP1 gene.. Proc Natl Acad Sci U S A.

[37] Turnley AM, Faux CH, Rietze RL, Coonan JR, Bartlett PF (2002). Suppressor of cytokine signaling 2 regulates neuronal differentiation by inhibiting growth hormone signaling.. Nat Neurosci.

[38] Rico-Bautista E, Greenhalgh CJ, Tollet-Egnell P, Hilton DJ, Alexander WS (2005). Suppressor of cytokine signaling-2 deficiency induces molecular and metabolic changes that partially overlap with growth hormone-dependent effects.. Mol Endocrinol.

[39] Harris J, Stanford PM, Sutherland K, Oakes SR, Naylor MJ (2006). Socs2 and elf5 mediate prolactin-induced mammary gland development.. Mol Endocrinol.

[40] Machado FS, Johndrow JE, Esper L, Dias A, Bafica A (2006). Anti-inflammatory actions of lipoxin A4 and aspirin-triggered lipoxin are SOCS-2 dependent.. Nat Med.

[41] Machado FS, Esper L, Dias A, Madan R, Gu Y (2008). Native and aspirin-triggered lipoxins control innate immunity by inducing proteasomal degradation of TRAF6.. J Exp Med.

[42] Rico-Bautista E, Flores-Morales A, Fernandez-Perez L (2006). Suppressor of cytokine signaling (SOCS) 2, a protein with multiple functions.. Cytokine Growth Factor Rev.

[43] Sovio U, Bennett AJ, Millwood IY, Molitor J, O'Reilly PF (2009). Genetic determinants of height growth assessed longitudinally from infancy to adulthood in the northern Finland birth cohort 1966.. PLoS Genet.

[44] Rasche A, Al-Hasani H, Herwig R (2008). Meta-analysis approach identifies candidate genes and associated molecular networks for type-2 diabetes mellitus.. BMC Genomics.

[45] Mizushima S, Nagata S (1990). pEF-BOS, a powerful mammalian expression vector.. Nucleic Acids Res.

